# The Mediterranean Diet as a Model of Sustainability: Evidence-Based Insights into Health, Environment, and Culture

**DOI:** 10.3390/ijerph22111658

**Published:** 2025-10-31

**Authors:** Pasquale Perrone, Loris Landriani, Roberta Patalano, Rosaria Meccariello, Stefania D’Angelo

**Affiliations:** 1 Department of Medical, Human Movement, and Well-Being Sciences (DiSMMeB), Parthenope University of Naples, 80133 Naples, Italy; pasquale.perrone@collaboratore.uniparthenope.it (P.P.); roberta.patalano@uniparthenope.it (R.P.); 2 Department of Business Administration and Economics, Parthenope University of Naples, 80133 Naples, Italy; loris.landriani@uniparthenope.it

**Keywords:** carbon footprint, Mediterranean diet, olive oil, polyphenols, sustainability, water footprint

## Abstract

The Mediterranean Diet (MD) is globally recognized not only for its well-established benefits to human health but also for its potential as a sustainable dietary model from environmental perspectives. Primarily based on plant-based foods, olive oil, fish, and seasonal and local products, the MD stands out for its ability to reduce overall mortality and the incidence of chronic diseases. At the same time, it is a low environmental impact dietary approach, contributing to the reduction in greenhouse gas emissions, water savings, biodiversity conservation, and soil regeneration. This narrative review was conducted by searching the Scopus and PubMed databases, covering all publications up to 2011, applying predefined inclusion and exclusion criteria, and ultimately including 33 studies. The paper provides a synthesis of the key elements that make the MD a paradigm of sustainability, analyzing critical indicators such as carbon, water, and energy footprints, land use, food waste generation, and carbon sequestration. It also addresses the decline in adherence to the MD, even in Mediterranean countries, highlighting the socio-economic, cultural, and behavioral causes, as well as the necessary strategies to promote its rediscovery and adaptation to contemporary contexts.

## 1. Introduction

The Mediterranean diet (MD) represents one of the most studied and globally recognized nutritional paradigms due to its benefits for human health and its potential as a model of environmental sustainability [1].

This dietary pattern, characterized by a high intake of plant-based foods, olive oil as the main source of added fat, and moderate consumption of fish and dairy products, has been shown to be associated with a significant reduction in all-cause mortality and in the incidence of cardiovascular, neurodegenerative, and oncological diseases and also showed positive effects on the gut microbiota [2,3,4,5,6].

Recognized by UNESCO as an Intangible Cultural Heritage of Humanity in 2013, the MD is not simply a dietary regimen but a complex system of skills, knowledge, rituals, and traditions related to the cultivation, harvesting, fishing, animal husbandry, preservation, processing, preparation, and consumption of food [7].

The scientific conceptualization of the MD has its roots in the pioneering studies conducted by American biologist Ancel Keys and chemist Margaret Keys, who first proposed this nutritional concept in 1975 [8]. The inspiration for this formulation came from the eating habits and culinary traditions typical of Crete, mainland Greece, and southern Italy, which were studied and documented in the early 1960s.

However, beyond its well-known nutritional benefits, the MD also represents a sustainable dietary model that aligns perfectly with global environmental and climate goals. This eating pattern promotes the responsible use of natural resources, helping to reduce the ecological footprint of the food system [9].

Evidence from epidemiological and environmental studies supports the benefits of MD. Meta-analyses of cohort studies have reported a 17–25% reduction in all-cause mortality and a 20–30% lower risk of cardiovascular disease among individuals with high adherence to the MD [10]. Additionally, life cycle assessments indicate that the MD can reduce dietary greenhouse gas emissions by 20–30% and water use by 15–25% compared with typical Western diets [11]. These findings provide quantitative evidence that the MD not only supports human health but also contributes to measurable environmental sustainability.

In a global context marked by growing concerns over food security, climate change, and pollution, the adoption and protection of MD can play a key role in simultaneously promoting human health and environmental sustainability [12]. Its food structure, which prioritizes local and seasonal ingredients, allows for a reduction in greenhouse gas emissions, water consumption, and soil degradation, crucial factors in an era when the global ecosystem is under pressure [13].

Climate change is, in fact, significantly worsening food insecurity and malnutrition. It negatively affects agricultural productivity, reduces labor ability in many regions of the world, and hampers access to essential resources such as clean water and sanitation services. Moreover, the effects of global warming severely disrupt food supply chains, causing interruptions in trade flows and making markets less stable [14].

Marine ecosystems are also undergoing profound changes: rising coastal water temperatures, decreasing oxygen levels, ocean acidification, and coral reef bleaching threaten marine biodiversity and undermine sustainable fishing, a key component of the MD [15]. In this context, preserving this dietary model means not only safeguarding a cultural heritage but also actively contributing to the construction of a more resilient, fair, and environmentally respectful food future.

Although the MD originated in Mediterranean countries, its principles have been adopted to varying degrees across the world. However, adherence to MD principles differs significantly between Mediterranean and non-Mediterranean populations due to cultural, economic, and environmental factors. In Mediterranean countries, traditional food habits, social eating practices, and the availability of local products facilitate adherence to MD patterns. Conversely, in non-Mediterranean countries, cultural preferences, higher costs of typical MD foods (such as olive oil, fresh fish, and seasonal produce), and the dominance of Westernized dietary models often limit adherence [16]. Economic disparities also play a crucial role: in some contexts, the MD may be perceived as a more expensive option, despite its long-term health and sustainability benefits [17]. These cultural and economic differences must be considered when promoting the MD globally, as they highlight the importance of context-specific strategies to improve accessibility, affordability, and cultural adaptation of Mediterranean dietary principles.

The purpose of this manuscript is to systematically evaluate the current evidence on the health, environmental, and cultural impacts of MD as a model of sustainable nutrition. Rather than proposing a new dietary model, this review aims to integrate findings from epidemiological, nutritional, and environmental studies to highlight the multidimensional benefits of the MD, identify gaps in adherence, and provide guidance for promoting its adoption in contemporary contexts.

## 2. Bioactive Components of the Mediterranean Diet

The MD is characterized as a dietary pattern predominantly based on plant-based foods, including daily consumption of whole grains, olive oil, fruits, vegetables, legumes, nuts, aromatic herbs, and spices [18]. Animal-based foods are consumed in smaller quantities, with a preference for fish and seafood as the main sources of animal protein. The nutritional structure of this dietary pattern recommends eating fish at least twice a week, while other animal proteins such as poultry, eggs, and dairy products (mainly cheese and yogurt) are consumed in smaller portions, either daily or a few times a week [19]. Red meat is limited to a few times a month, marking a distinctive feature compared to conventional Western dietary patterns [20].

Olive oil is the cornerstone of the MD, recommended as the primary added fat in place of other oils and fats such as butter and margarine. This choice is not arbitrary, as olive oil has been the subject of numerous scientific studies highlighting its potential protective role in reducing all-cause mortality and the risk of chronic diseases [21]. Extra virgin olive oil (EVOO), in particular, is rich in monounsaturated fatty acids, mainly oleic acid, which favorably modulate plasma lipid profiles by lowering levels of oxidized LDL cholesterol and increasing HDL cholesterol [22]. Additionally, it is a significant source of polyphenols (e.g., oleuropein, hydroxytyrosol), which exert antioxidant, anti-inflammatory, and vasoprotective effects at the endothelial level, contributing to the reduction in systemic oxidative stress and chronic low-grade inflammation [23,24,25,26]. Numerous epidemiological and clinical studies suggest that a high intake of EVOO is associated with a reduced risk of major cardiovascular events such as myocardial infarction and ischemic stroke, as well as a lower risk of cognitive decline and dementia [27,28,29,30,31]. Its phenolic compounds also affect gene expression and modulate sirtuin activity, thereby contributing to the slowing of cellular aging processes [32].

Other foods naturally rich in healthy fats are also emphasized in the Mediterranean pattern, including avocado, nuts, and fatty fish such as salmon and sardines. Among these, nuts and fish are particularly valued for their high content of omega-3 fatty acids, bioactive compounds with well-documented anti-inflammatory and cardioprotective properties [33]. Long-chain polyunsaturated fatty acids like EPA and DHA, abundant in fatty fish, positively influence endothelial function, modulate eicosanoid production, and play a crucial role in neuroprotection and reducing the risk of neurodegenerative diseases [34]. Omega-3s are also involved in regulating the activity of nuclear receptors such as PPAR and in intracellular signal transduction, helping reduce inflammatory responses and improve insulin sensitivity [35]. Regular dietary intake of omega-3s has been associated with lowered blood pressure, improved post-infarction cardiac function, and stabilization of atherosclerotic plaques [36].

The plant-based part of MD is rich in bioactive compounds, particularly antioxidants, which contribute significantly to its health-promoting effects [37,38]. Typical plant foods in this dietary pattern, including fruits, vegetables, unprocessed grains, legumes, and herbs, contain a multitude of antioxidants capable of neutralizing free radicals [39,40,41]. These bioactive compounds include carotenoids, which not only contribute to vitamin A intake but also have potent antioxidant properties. The lipophilic nature of carotenoids makes their co-ingestion with fats optimal, perfectly aligned with the use of olive oil in the MD [42]. Other important phytochemicals include flavonoids, found in citrus fruits, onions, grapes, and red wine, which exhibit anti-inflammatory, antitumor, and antiatherogenic activities by inhibiting pro-inflammatory signaling pathways such as NF-κB and MAPK [43,44]. The high phytonutrient density of Mediterranean plant foods also promotes metabolic homeostasis and mitochondrial health, helping reduce the risk of insulin resistance, visceral obesity, and metabolic dysfunction [45]. Phenolic compounds and fermentable fibers support a eubiotic gut microbiota composition, enriching beneficial taxa like Bifidobacterium and *Faecalibacterium prausnitzii* and decreasing intestinal permeability associated with chronic inflammatory states [5,46]. Furthermore, dietary fiber from legumes, fruits, and vegetables promotes gut health by enhancing microbiota diversity, increasing production of short-chain fatty acids (SCFAs) with anti-inflammatory effects, and contributing to the regulation of postprandial blood glucose and insulin sensitivity [47].

It is important to note that while cereals and other carbohydrate-rich foods traditionally occupy the base of the Mediterranean food pyramid, recent research in metabolic health and personalized nutrition suggests that carbohydrate quality and quantity should be tailored to individual metabolic profiles [48]. Whole grains remain a key source of fiber, vitamins, and bioactive compounds, but the proportion and frequency of their consumption may need to be adapted according to energy needs, glycemic response, and metabolic status [49]. This more flexible interpretation aligns the Mediterranean model with current evidence on individualized dietary approaches.

The choice of water as the main daily beverage is another defining feature, with the possibility of moderate wine consumption during meals, approximately one to two glasses per day for men and one glass per day for women. This recommendation reflects the balanced approach of MD, recognizing the cultural role and potential health benefits of moderate wine consumption without encouraging alcohol abuse. Red wine, in particular, is a source of resveratrol and other polyphenols, which, when consumed in moderation, have been associated with reduced cardiovascular risk through antioxidant mechanisms and improved vascular function [50]. Resveratrol has also been shown to activate metabolic pathways involved in cellular longevity, such as sirtuins and AMPK, and to exert neuroprotective effects through the modulation of neuroinflammation and oxidative stress [51,52]. However, it is important to emphasize that such benefits are evaluated only with moderate consumption and as part of an overall healthy lifestyle. Nevertheless, current scientific consensus and public health authorities, including the World Health Organization, emphasize that no level of alcohol consumption can be considered completely safe for health [50,53]. The reference to wine within MD should therefore be interpreted primarily in its cultural and traditional context, rather than as a recommendation for consumption [54]. In modern dietary guidelines, the focus remains on the promotion of non-alcoholic beverages, particularly water, herbal infusions, and natural fruit-based drinks, as the healthiest hydration choices within the Mediterranean model [55].

**Figure 1:**
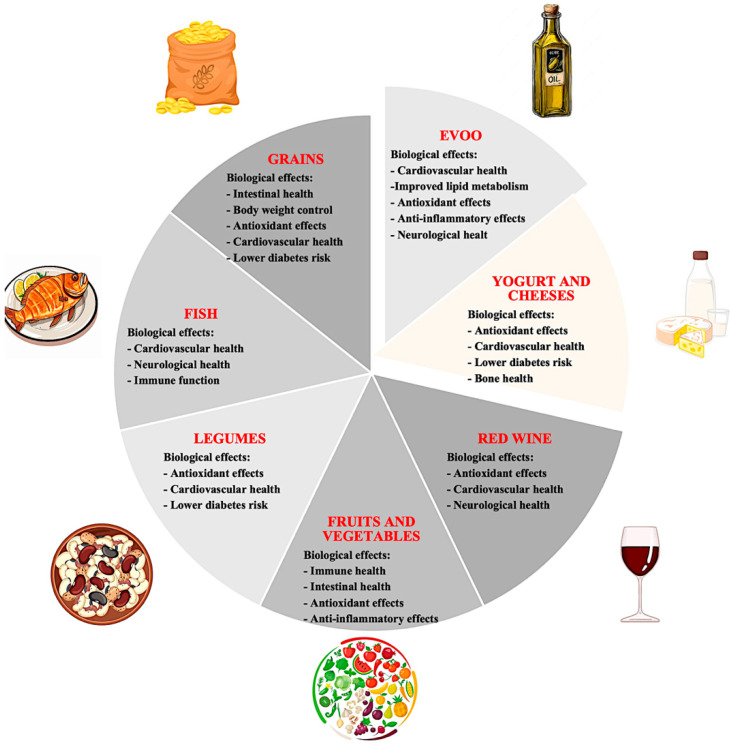
Main components of the Mediterranean diet and key health benefits.

The emphasis on daily physical activity through enjoyable routines completes the lifestyle picture, highlighting how MD represents a holistic approach to wellness that goes beyond mere nutritional composition [23,56,57,58,59,60,61]. This integrated approach has been shown to positively modulate systemic inflammatory markers (e.g., CRP, IL-6, TNF-α), contributing to the primary prevention of non-communicable chronic diseases such as cardiovascular disease, type 2 diabetes, metabolic syndrome, and certain cancers [62,63,64,65,66,67]. Numerous longitudinal studies and randomized clinical trials, such as the PREDIMED study, have confirmed the effectiveness of MD in reducing the incidence of major cardiovascular events, improving cognitive function, and lowering the risk of depression [68,69]. The synergistic integration of nutrients, phytochemicals, and healthy behaviors characteristic of this dietary model provides a sustainable metabolic advantage adaptable to diverse populations (Figure 1).

Although MD is often treated as a monolithic concept, it is important to highlight that there are significant geographical variations within the Mediterranean basin itself, which can affect both its health and environmental outcomes. These variations are influenced by local food production systems, cultural practices, and socioeconomic conditions, all of which can alter the composition of the diet. For instance, the dietary patterns in southern Italy, Greece, and Spain may differ in terms of the consumption of plant-based foods, types of animal products, and processing methods, which can lead to different environmental impacts, such as water footprint, carbon emissions, and land use. Moreover, even within individual countries, such as Italy or Spain, regional variations may arise due to local agricultural practices, availability of specific foods, and traditional culinary habits.

For example, while the typical MD emphasizes olive oil, legumes, fruits, and vegetables, the ecological footprint of these foods can vary depending on local production methods (e.g., the use of irrigation or organic farming techniques) and seasonality. These geographic differences must be considered to fully understand the sustainability and health impacts of the MD and to ensure that policy recommendations reflect the diversity of local contexts. Additionally, the socio-economic factors in different regions, such as income disparities and food access, also contribute to variations in adherence to MD and its sustainability outcomes [70,71].

## 3. Methodology

**Figure 2:**
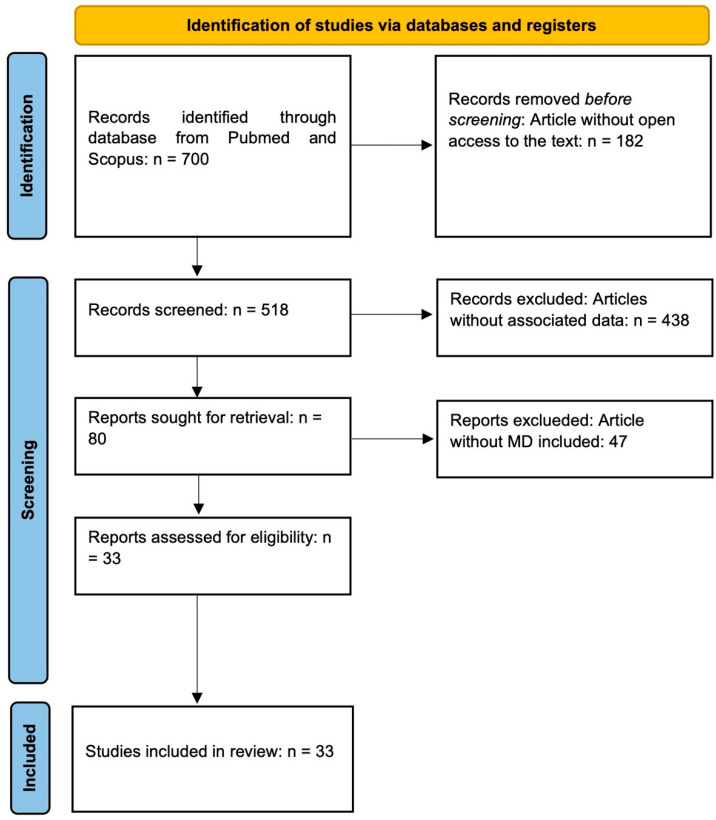
Flow diagram of the literature selection process. The structure is inspired by the PRISMA framework but adapted to the narrative review methodology. Studies were retrieved from SCOPUS and PubMed using the keywords ‘Mediterranean Diet’ and ‘Sustainability.’ Articles not directly addressing the Mediterranean Diet or without full-text access were excluded. The remaining studies were included in the final analysis.

This review is structured as a narrative synthesis of the literature, incorporating certain systematic elements such as database searches and predefined inclusion criteria. However, it does not adhere to the full PRISMA 2020 guidelines, which require a registered protocol, a comprehensive search strategy, detailed inclusion/exclusion criteria with justification, a risk of bias assessment, and a standardized flow diagram. Therefore, the present work should be considered a narrative review with structured components, rather than a systematic review. The flow diagram included (Figure 2) is inspired by the PRISMA framework and is intended to provide transparency in the selection process, but it does not imply full compliance with PRISMA standards.

A narrative review of the literature on the environmental sustainability of the MD was conducted. The databases consulted included SCOPUS and PubMed (MEDLINE). Articles were selected based on title, year of publication, review of abstracts, and reading of the full text to assess relevance. Keywords used in the search included a combination of the terms “Mediterranean Diet” and “Sustainability”. Articles without access to the full text were not included in the final analysis. This exclusion was applied for reasons of methodological transparency and reproducibility, as the research team had limited institutional access to paywalled publications at the time of the review. Consequently, only studies whose full texts were freely accessible were analyzed, ensuring that all data considered could be independently verified by readers. Studies were included and selected based on their relevance in associating the MD with improved environmental sustainability (Figure 2).

## 4. The Mediterranean Diet as a Paradigm of Environmental Sustainability

Although broad and transversal, over the past ten years, the topic of sustainability has been considered central in economic and business studies, also at an international level. This is shown by the growing number of studies published in leading sector journals. This increasing interest reflects the need to integrate traditional economic logics with a broader and multidimensional vision that considers sustainability as a strategic lever for innovation and the resilience of production systems.

It is worth remembering that this is not a new topic but rather a cyclical return, which first emerged in the early 1960s and was further developed in the 1990s [72]. In particular, the introduction of the triple bottom line concept marked a turning point in the approach to corporate responsibility, promoting a balance between economic performance, environmental protection, and social well-being.

The origins of these studies lie initially in environmental awareness, then extended to social issues, and finally to the interactions and synergies with classical economic levers, although their integration remains controversial and debated in scientific literature. The main challenge is to overcome the dichotomy between economic growth and sustainability by showing models that are simultaneously competitive, inclusive, and respectful of ecological limits.

In this perspective, a crucial context is certainly the agri-food sector, as it is used in direct symbiosis with nature while also having primary economic and social importance. The agri-food sector is indeed characterized by intense interrelations with the environment, human labor, public health, and local cultural dynamics [73].

Currently, agriculture is likely one of the most important contributors to sustainable development for the planet, people, and the economy. It amplifies the links between environmental protection, climate change, natural resource exploitation, and social equity, assigning new responsibilities to companies within the supply chain and beyond. Modern agriculture, in particular, stands at the crossroads between the need to guarantee food security and the urgency to reduce environmental impact while promoting equitable and resilient production models [74].

The growing concern over the environmental impact of global food systems has led to renewed interest in MD as a model of sustainable consumption. This dietary pattern has been recognized as sustainable due to its lower environmental impact on land use, water, and energy, as well as its positive potential for climate mitigation. According to the FAO and CIHEAM, the MD represents an intangible cultural heritage that combines health, environmental sustainability, and local identity, offering a concrete response to the challenges of ecological and food transition [75].

Scientific research has documented that the MD has significantly lower environmental footprints compared to the traditional Western Diet, demonstrating this dietary model’s potential to contribute to the sustainability of agri-food systems [76]. Moreover, the ratio between plant and animal proteins represents a key indicator of food sustainability, and MD excels in this parameter. A study conducted in France as part of the NutriNet-Santé Study showed that MD combined with organic foods presents the best ratio between plant and animal proteins. For conventional MD followers, this ratio was 0.66, while for those following an organic MD, the ratio rose to 1.38, indicating a higher proportion of plant proteins [77]. This aspect is crucial for sustainability, as the production of plant proteins generally requires fewer resources and generates lower greenhouse gas emissions than animal protein production. This proportion also favors greater efficiency in the use of natural resources and less pressure on agricultural ecosystems.

In general, the MD is configured as a sustainable dietary model from multiple environmental, economic, social, and health perspectives. Its structure, based on the predominant consumption of fresh, local, and seasonal plant foods, combined with moderation in the use of animal products, makes it compatible with global sustainability goals [9]. Furthermore, it promotes a healthy lifestyle, a lower ecological impact, fair income distribution within the agricultural supply chain, and the strengthening of rural economies [78].

However, the evaluation of MD impact cannot ignore a critical and detailed reading of the various parameters that determine its sustainability. Indeed, the intrinsic complexity of food systems requires a multidimensional analysis capable of capturing not only environmental outcomes (such as greenhouse gas emissions, land use, energy, and water consumption) but also social, cultural, and economic implications. A systemic and integrated approach is indispensable to understanding the interconnections among different sustainability indicators and to avoiding partial or misleading assessments.

Therefore, it is necessary to continue with a systematic and detailed review of each parameter, such as water footprint, carbon footprint, energy consumption, food waste, impact on biodiversity, and carbon sequestration ability, to offer a comprehensive and scientifically grounded evaluation of the Mediterranean model. Such analysis allows the identification of strengths as well as potential criticalities or contradictions, such as the higher water footprint associated with certain plant foods or the higher economic costs of a high-quality diet [79]. These aspects must be analyzed in the context of geographical, socioeconomic, and cultural specificities to guide effective and sustainable policy interventions. Only through an integrated and evidence-based approach is it possible to precisely define the real contribution of MD to the sustainability of food systems, as well as to enhance its potential as a tool for food and environmental policy, even in geographic contexts beyond the Mediterranean basin. Its adaptability to different cultural and productive contexts is a strategic opportunity to promote sustainable food transitions on a global scale.

### 4.1. Carbon Footprint

The carbon footprint is the total amount of greenhouse gases, expressed in kg of CO_2_ equivalent (CO_2_eq), generated throughout the entire life cycle of food: from primary production, industrial processing, and transportation to final consumption and waste management. This metric is a key indicator for assessing the environmental impact of different food choices and supporting policies aimed at the sustainability of the agri-food system [80].

Numerous studies have shown that MD is characterized by significantly lower emissions compared to Western dietary models with high consumption of red meat, saturated fats, and ultra-processed products [81]. Specifically, a systematic analysis of the sustainability dimensions of MD revealed that this dietary pattern has a carbon footprint ranging from 0.9 to 6.88 kg CO_2_eq per person per day, with an average around 4 kg CO_2_eq/day, values substantially lower than the conventional Western diet, which can exceed 7–9 kg CO_2_eq/day [79].

An analysis conducted in Spain calculated that the daily ecological footprint of the MD is about 5.08 kg CO_2_eq per person, compared to 7.4 kg for a typical English diet and 8.5 kg for a U.S. diet, with equal caloric intake (about 2000 kcal) [82]. This finding was further corroborated by a European meta-analysis estimating, for the ideal MD, emissions around 2.3 kg CO_2_eq per person per day, consistent with the global climate targets outlined by the IPCC. However, emissions associated with diets actually followed in Mediterranean countries are higher (up to 4.46 kg CO_2_eq/day), due to the growing adoption of hybrid eating habits that include excessive amounts of meat and refined products, deviating from the traditional model [83].

The main factor in emission differences is red meat consumption. It is estimated that simply reducing one weekly meat meal, replaced with a typical Mediterranean plant-based dish (e.g., legumes or whole grains), can save about 180 kg of CO_2_eq per person per year [84]. Seasonality and locality of plant products also strongly affect emissions: 1 kg of tomatoes grown out of season in a greenhouse can emit up to 3.5 kg CO_2_eq, compared to less than 0.05 kg for the same amount grown in open fields during summer [85]. Therefore, thanks to lower meat intake and a high presence of local, seasonal plant foods, MD enables a reduction in greenhouse gas emissions between 30% and 50% compared to Western dietary models [81]. This reduction is particularly relevant in the context of the 2030 Agenda for Sustainable Development and the climate neutrality goals set for 2050. Of particular interest is also the association between adherence to MD and the mitigation of emissions from food production, which accounts for about one-third of total global greenhouse gas emissions. Intensive livestock farming, in particular, handles high methane (CH_4_) levels, a greenhouse gas with a global warming potential 28 times greater than CO_2_. MD's emphasis on plant-based foods, combined with moderate consumption of animal products, especially red meat, results in significantly lower carbon emissions compared to Western dietary models [86].

Dietary data from the National Health and Nutrition Examination Survey (NHANES 2011–2018) were integrated with environmental impact data, agricultural resource demands, and food prices extracted from many public databases. The aim was to assess the association between adherence to the Mediterranean dietary model and various sustainability indicators. The study highlighted that greater adherence to the MD correlates with reduced greenhouse gas emissions, land use, and fertilizer and pesticide application, although it is associated with an increased water footprint and higher overall diet cost [87]. This underscores the need for holistic approaches that integrate different dimensions of food sustainability to avoid environmental and social trade-offs.

Many studies have proved that diets rich in red meat can generate up to five times more greenhouse gas emissions than predominantly plant-based diets. MD, based on legumes, whole grains, nuts, seeds, and fish from sustainable fishing practices, helps reduce these emissions [88]. The selective and moderate inclusion of animal proteins, especially from fish and low-impact local dairy products, further contributes to reducing the overall environmental impact [84].

Conversely, intensive livestock farming is responsible not only for high methane emissions but also for deforestation, as vast areas of land are converted for grazing or feed crop cultivation. This process negatively affects biodiversity, alters biogeochemical cycles, and compromises ecosystem resilience. These cumulative effects position food as a critical junction for ecological transition [89].

By promoting sustainable food choices, MD significantly contributes to mitigating climate change. Mediterranean culinary traditions, which make extensive use of seasonal and locally produced ingredients, further reduce the carbon footprint related to transportation and food preservation. Encouraging the consumption of locally grown fruits and vegetables, alongside reducing the use of highly processed and packaged foods, strengthens the environmental benefits of this dietary model. Additionally, the low content of imported or energy-intensive foods helps reduce the energy needed for refrigeration and logistics [86]. This approach supports ecological sustainability, enhances food sovereignty, and reinforces local agricultural economies, offering a scalable model for agri-food policies in climatically and culturally comparable regions.

### 4.2. Water Footprint

The water footprint (WF) measures the total volume of freshwater used to produce consumed foods, including three main components: “green” water (rain incorporated in agricultural products), “blue” water (withdrawn from surface or groundwater sources for irrigation), and “grey” water (the amount needed to dilute pollutants generated during production). This indicator is essential for assessing the water impact of food systems and their long-term sustainability [90].

MD stands out for significantly lower water use compared to other dietary regimes, thanks to the predominance of plant foods and reduced reliance on water-intensive resources. According to a CREA study, the MD saves about 25% of water compared to other diets, with an average saving of 1104 L of water per person per day. Annually, this equates to a reduction of over 400,000 L per individual, with significant environmental impacts in water-stressed contexts [91].

Analyzing Italian data, it is observed that for those adhering strongly to MD, the daily water footprint is about 3243 L per capita, compared to much higher values for those regularly consuming meat and animal derivatives [92]. Internationally, adoption of MD in Mediterranean Basin countries reduces the food–water footprint by 4% to 35%, depending on adherence levels and regional variants [91].

Water consumption reduction is attributable to the prevalence of cereals, legumes, fruits, and vegetables, which have a lower water footprint compared to meat and dairy; the seasonality of products; and less reliance on intensive irrigation systems. Specifically, beef production requires up to 15,000 L of water per kg, while legumes require less than 4000 L per kg. Traditional Mediterranean crops, such as olives and vines, show high water efficiency, further contributing to system sustainability [93]. However, it is important to acknowledge that not all plant-based foods typical of MD have a low water footprint. Certain high-value products, such as nuts (especially almonds and pistachios), as well as some fruits, require substantial amounts of irrigation water, particularly when cultivated in arid or semi-arid regions. This highlights that, while the overall water efficiency of the Mediterranean model remains favorable compared to animal-based diets, significant intra-diet variability exists [94,95]. Recognizing these trade-offs is essential for a nuanced understanding of the environmental implications of MD and for guiding more sustainable production and consumption choices.

Preserving water resources is one of the most urgent environmental challenges globally today. Increasing anthropogenic pressure, climate change, and groundwater pollution compromise the availability and quality of freshwater. Adopting low-water-impact dietary models, like MD, helps mitigate water scarcity, which increasingly affects arid and semi-arid regions of the planet, including many Mediterranean Basin areas [96].

The water efficiency of MD is closely linked to the promotion of sustainable agricultural practices, such as crop rotation, agroecology, and the use of drought-resistant crops. These strategies not only reduce direct water use for irrigation but also improve soil moisture retention capacity, lowering the overall water needs of production systems [97]. Such integrated approaches can enhance the climate resilience of agricultural territories and reduce vulnerability to prolonged droughts.

From a food security perspective, reducing the water footprint is a strategic goal to ensure the resilience of the agri-food system to water crises, avoid conflicts over resource access, and promote a more equitable and responsible distribution of water. Water, being a limited public good, requires sustainable management strategies that also engage consumers through conscious choices.

Finally, food education and consumer awareness play a fundamental role in reducing the water impact of the diet. Choosing local, seasonal, and plant-based products not only helps safeguard natural resources but also supports sustainable and circular agricultural economies. Integrating water footprint information into nutritional labels and educational programs could be an effective tool to guide individual choices toward more sustainable consumption.

### 4.3. Food Waste

Food waste is one of the main environmental challenges of modern food systems, as it leads to the waste of natural resources (water, soil, and energy) and an increase in greenhouse gas emissions. MD, thanks to its traditional practices and the valorization of leftovers, is associated with a significant reduction in food waste [98].

A cross-sectional study conducted on Italian university students showed that greater adherence to MD is correlated with more virtuous behaviors in food and leftovers management, with a halved probability of producing high levels of waste (Odds Ratio = 0.50). Those who follow MD demonstrate better skills in planning purchases, managing quantities, and reusing leftovers, also thanks to the positive influence of family habits and a culture of recovery [99].

This relationship is also supported by qualitative analyses conducted in Mediterranean domestic contexts, where high food literacy and the value attributed to home cooking promote optimal stock management and reduce overproduction [100].

Moreover, the MD favors the consumption of fresh, local, and seasonal foods, which tend to have a shorter shelf life but are consumed quickly, reducing the risk of spoilage and waste. This characteristic, combined with more attentive and home-based meal preparation, helps avoid excessive reliance on packaged and ultra-processed foods, which often have higher discard rates throughout the supply chain, from production to final consumption [101].

Another distinctive element of MD is the intergenerational transmission of recipes and traditional culinary practices that include, among others, the creative reuse of leftovers. Several typical Mediterranean dishes transform “waste” into new preparations. This cultural approach results in a more resilient food system that respects natural resources. Reducing food waste is not only an environmental issue but also an ethical and economic one: every wasted food item is a loss for producers, sellers, and buyers alike. In a world where millions suffer from hunger, containing waste also means contributing to a fairer and more equitable use of resources [102].

From a circular economic perspective, MD can thus be a sustainable dietary model also for the prevention and management of waste, thanks to a combination of conscious individual choices, gastronomic culture, food education, and community practices. Integrating awareness-raising strategies about the value of food, educational programs in schools, and public policies aimed at reducing waste along the entire supply chain (from production to consumption) can further strengthen the effectiveness of MD as a lever for systemic sustainability.

### 4.4. Energy Consumption

Energy consumption associated with the production, processing, and transportation of food is another crucial aspect of sustainability. MD, thanks to its structure based on plant foods and short supply chains, requires less energy compared to dietary models high in animal products and ultra-processed foods [103].

Comparative studies have estimated that the annual energy requirement to support an MD is about 5250 MJ per person, which is 28% lower than that of a standard Western diet. This saving derives from less industrial food processing, reduced need for refrigeration and long-distance transportation, and the prevalence of fresh and minimally processed foods [81].

Meat production, particularly beef, is among the most energy-intensive activities in the entire food system, requiring energy for livestock farming, processing, and preservation. MD, by limiting red meat consumption and favoring plant protein sources and fish, substantially contributes to reducing total energy consumption [20].

At the product life cycle level (Life Cycle Assessment), differences are significant: producing 1 kg of beef can require up to 70 MJ of energy, compared to about 10–15 MJ for the same number of legumes. These data highlight the potential for energy reduction through a transition to more plant-based dietary models [104].

It is important to note that not all red meat contributes equally to environmental and health impacts. Evidence suggests that moderate consumption of lean, unprocessed red meat, particularly from extensive or pasture-based systems, can be compatible with healthy dietary patterns and has a lower ecological footprint compared to heavily processed meats or intensively farmed beef [105,106]. Life cycle assessments show that pasture-raised beef generally requires less energy per unit of protein when compared to industrial feedlot systems, due to reduced inputs in feed production and lower reliance on energy-intensive confinement operations [107,108]. Therefore, promoting a Mediterranean dietary pattern does not necessarily imply the complete elimination of red meat but rather encourages moderation, selection of unprocessed and sustainably produced options, and prioritization of plant-based proteins, in line with both nutritional and environmental objectives.

Additionally, the use of simple and traditional cooking methods, such as boiling, grilling, and baking, typical of MD, involves less energy expenditure compared to the complex processes required by industrial prepackaged dishes. Home meal preparation, a central element of Mediterranean culture, not only strengthens the connection with the territory and seasonality but also helps reduce the energy footprint associated with the food industry.

Another often overlooked but fundamental aspect concerns short supply chains and reduced transportation. The traditional MD relies on local and seasonal products, which do not require long transport or prolonged storage. This results in a significant reduction in emissions linked to logistics and packaging [109].

Air transportation of food products, for example, can drastically increase CO_2_ emissions: strawberries, apples, tomatoes, and other vegetables transported across continents have a much higher impact than the same products consumed locally and in season. By encouraging local consumption, MD thus helps minimize the ecological footprint of transport.

Added to this is the positive effect of the low energy density of MD, which implies a lower caloric need per unit of weight, indirectly contributing to reduced energy use for food production.

It should also be emphasized that transitioning to lower-energy-intensity dietary models is one of the strategies recommended by major international agencies to tackle the climate crisis. In this context, MD represents an already available and tested response, capable of combining human health, taste, culture, and energy savings [110].

Promoting MD, therefore, means not only improving the population’s dietary habits but also contributing to building a more efficient, resilient, and ecologically respectful food system. From a systemic perspective, large-scale adoption of MD could generate significant energy savings at national and community levels, contributing to the decarbonization of the agri-food sector and the achievement of climate neutrality goals.

### 4.5. Land Use

Land use is a fundamental indicator of sustainability, as the conversion of natural lands into agricultural areas is one of the main causes of biodiversity loss, erosion, and desertification. MD, thanks to its predominantly plant-based food structure, requires less agricultural land compared to diets high in meat [109].

According to Spanish data, MD involves agricultural land use of about 2000 square meters per person per year, a value 40% lower than the Western diet rich in meat and dairy products. This result is attributable to the greater efficiency of plant crops (cereals, legumes, and vegetables) compared to the production of fodder and feed for animal farming [111].

Crops intended for direct human consumption, such as legumes or whole grains, require less land, energy, and natural resources per unit of protein produced compared to meat. Unlike intensive livestock farming, which needs large areas for feed production, Mediterranean plant production optimizes land yield and reduces pressure on natural ecosystems [89].

In terms of territorial efficiency, it is estimated that obtaining 100 g of protein from animal sources requires up to 10 times more agricultural land than from plant sources. This difference amplifies the impact of dietary choices on land use and the carrying capacity of agricultural ecosystems [93].

Moreover, MD promotes crop rotation, polyculture, and the use of local varieties, practices that help support soil fertility, prevent erosion, and enhance the resilience of agricultural ecosystems. These agricultural techniques, deeply rooted in Mediterranean rural tradition, counteract land degradation and support the natural regeneration of soils. These traditional practices integrate with the principles of agroecology and regenerative agriculture, promoting a multifunctional use of land that combines food production, ecosystem services, and landscape protection.

Finally, the integration of tree crops such as olive groves, orchards, and vineyards, typical of the Mediterranean agricultural landscape, contributes positively to soil structure, atmospheric carbon capture, and biodiversity conservation. These agroforestry systems promote ecological stability, create microhabitats for local fauna, and play a key role in mitigating the environmental impacts of agriculture [112]. In particular, the olive tree is considered a strategic crop for carbon sequestration in Mediterranean soils, thanks to its extensive root system and traditional management with spontaneous vegetation cover.

Therefore, promoting MD not only has direct benefits for human health but also is an effective tool to reduce human pressure on territorial resources and promote a regenerative agricultural model capable of preserving natural capital for future generations.

### 4.6. Biodiversity and Ecosystem Conservation

Biodiversity is one of the key elements of food sustainability. MD, based on a great variety of native plants and animal species, actively supports the conservation of agricultural and natural biodiversity.

In Mediterranean countries, over 4800 plant varieties are cultivated, and hundreds of local animal breeds are raised, many of which are at risk of extinction in intensive agricultural systems. The variety of crops (wheat, barley, legumes, olive, vine, and vegetables) and traditional farming techniques (rotations, intercropping, and terraced cultivation) help maintain rich and diverse habitats, also favoring the presence of pollinators and other organisms beneficial to ecosystem health [113].

The polyculture and diversified approach of MD contrasts with the trend towards intensive monoculture, the main cause of ecological simplification and loss of resilience in agroecosystems. The use of local varieties selected over time to adapt to specific pedoclimatic conditions reduces the need for external inputs such as fertilizers and pesticides [87]. These agricultural systems, passed down through generations, create mosaic agrarian landscapes capable of hosting a multitude of plant and animal species, reducing vulnerability to diseases, pests, and climate change. In this way, ecological stability and territorial resilience are promoted. By valuing seasonal and local products, MD encourages the conservation of traditional varieties and agricultural knowledge, countering the standardization and genetic erosion typical of industrial agriculture.

The recovery and protection of local cultivars (such as San Marzano tomato, violet artichoke, or Castelluccio lentil) and native breeds (such as Sardinian sheep or Casertano black pig) not only strengthen local food security but also create economic opportunities through short supply chains and quality certifications. Promoting the consumption of products derived from native breeds and ancient varieties also means supporting local rural communities, safeguarding agricultural and food genetic heritage, and preserving the cultural memory of entire territories.

Moreover, food biodiversity is closely linked to nutritional diversification: a diet rich in variety guarantees a better supply of micronutrients, antioxidants, and bioactive compounds [114]. Maintaining a wide range of species and cultivars is therefore not only an environmental issue but also a crucial factor for public health [115].

In a global context where few species dominate world food production (mainly wheat, maize, rice, and soy), the Mediterranean food model is a concrete alternative to safeguard genetic and biological diversity while promoting sustainability, food security, and human well-being.

Strengthening the connection between biodiversity conservation and traditional food models like MD is now a key strategy recognized also by international organizations such as FAO and the European Commission, aligned with the 2030 Agenda for Sustainable Development.

In this context, the use of wild sources of nutrients, particularly wild edible plants, herbs, and spontaneous greens, represents a further dimension of Mediterranean biodiversity and sustainability. These species, traditionally gathered and consumed in rural communities, provide low-cost and nutrient-rich food options that require minimal external inputs, cultivation, or transportation [116]. Their use reinforces local food autonomy and supports ecosystem services by preserving marginal habitats and traditional ecological knowledge [117]. However, foraging practices must be managed responsibly: misidentification of toxic species, harvesting in contaminated areas, and overcollection can pose health and environmental risks. Ensuring food safety and ecological balance requires education, monitoring, and preservation of ethnobotanical knowledge transmitted through generations. Furthermore, the cultural specificities related to the recognition, preparation, and culinary use of wild edible plants across Mediterranean regions reflect an invaluable heritage that integrates biodiversity, food security, and cultural identity within the MD framework [118].

### 4.7. Carbon Sequestration and Soil Regeneration

Agricultural practices associated with MD, such as the use of tree crops (olive, vine, citrus) and traditional soil management (green manure, organic fertilization, grass cover), play a crucial role in combating climate change by promoting carbon sequestration in the soil [97].

It is estimated that Mediterranean agricultural systems can sequester up to 2.4 O of CO_2_ equivalent per hectare per year, thus contributing concretely to the mitigation of climate-altering emissions. This outcome is made possible by the soil’s capacity to store organic carbon through plant photosynthesis and the subsequent transfer of biomass into the soil in the form of roots, crop residues, and stable organic matter (humus) [119].

Soil organic carbon (SOC) is one of the main forms of terrestrial carbon storage, with a mitigation potential of about 10% of global greenhouse gas emissions if properly managed. In particular, perennial tree crops such as olive and vine, thanks to their long lifespan and permanent presence in the field, are able to accumulate significant amounts of carbon both in above-ground biomass and in the soil, making these production systems true natural “carbon sinks” [120].

The presence of trees, hedges, and traditional agricultural landscape elements, such as dry-stone walls and terraces, typical of Mediterranean countryside, not only increases the ability to absorb atmospheric CO_2_ but also performs a fundamental function in protecting the soil from erosion, regulating the microclimate, and reducing evaporation. This contributes to improving the resilience of agricultural systems to extreme climatic events such as droughts and heavy rainfall [119]. Moreover, these traditional practices increase soil porosity, enhance water infiltration, and stimulate beneficial microbial activity (bacteria and mycorrhizal fungi), which are key elements in the long-term regeneration of soil fertility.

The agroecological approach characterizing many MD practices does not merely conserve soil but promotes its biological activation and regenerative capacity, reducing dependence on chemical inputs and sustainably increasing productivity [121].

Integrating these practices into broader agricultural models, even outside the Mediterranean context, could be a replicable and low-cost strategy to contribute to global decarbonization and climate adaptation goals, as outlined in the Paris Agreement and the EU’s “Farm to Fork” Strategy, thereby strengthening agriculture’s role as a lever for ecological transition.

### 4.8. Social and Cultural Sustainability

The sustainability of MD extends beyond purely environmental aspects, embracing social, economic, and cultural dimensions in an integrated perspective consistent with the systemic vision promoted by the United Nations 2030 Agenda. According to this approach, food systems must be not only ecologically efficient but also equitable, inclusive, resilient, and respectful of cultural diversity [122].

The recognition of MD as Intangible Cultural Heritage of Humanity (UNESCO, 2010) highlighted how this food model is closely linked to social practices such as hospitality, conviviality, intergenerational dialog, and community cooperation. The meal is not just a nutritional act but a collective experience that strengthens social and family bonds, promotes mental health, and fosters a sense of belonging [7].

MD plays a fundamental role in cultural spaces, festivals, and celebrations, bringing together people of all ages and backgrounds, contributing to social cohesion and the resilience of local communities. In an era marked by increasing individualism, disintegration of family networks, and urbanization, reclaiming communal moments related to food becomes an act of cultural resistance and reclaiming everyday life.

Intergenerational transmission of knowledge, especially by women, is a cornerstone of the cultural sustainability of MD. Women preserve and pass on knowledge about culinary techniques, seasonality, the use of wild herbs, food preservation, and processing, playing a fundamental role in the cultural reproduction of the food system.

Traditional markets are key spaces for the dissemination and daily practice of MD. Places of both economic exchange and social interaction, markets ease access to fresh, local, and seasonal products, strengthen trust relationships between producers and consumers, and keep alive the agri-food memory of territories. They also serve as informal educational environments where people learn to recognize quality, seasonality, and the value of food.

Supporting these short and relational distribution circuits is essential to strengthening food sovereignty, countering the standardization imposed by large-scale distribution, and reducing dependence on often less fair and more impactful global supply chains.

MD also contributes to economic sustainability by promoting small-scale, multifunctional, and territory-linked agriculture. Supporting small producers, food artisans, and family businesses favors the vitality of rural areas, economic diversification, and the enhancement of food and wine tourism, with benefits in employment, income, and protection of the agrarian landscape [123].

In summary, MD is a systemic and regenerative food model that integrates health, environment, culture, and social justice, standing for a concrete and sustainable alternative to face the interconnected crises of our time (Table 1 and Table 2).

## 5. Structural Limitations of the Mediterranean Model

Although MD is widely recognized by the scientific community as one of the most favorable dietary models for human health and environmental sustainability, a rigorous and multidimensional evaluation requires the identification of several structural and operational limitations that may constrain its full applicability or partially compromise its consistency with integrated sustainability principles (Table 3).

Firstly, although adherence to MD is generally associated with a reduced water footprint compared to Western dietary patterns, certain plant-based foods typical of the model, such as greenhouse-grown tomatoes, almonds, and avocados, show high water demand, particularly in contexts characterized by water scarcity and intensive agriculture. The elevated water usage associated with the production of these crops, if not managed in accordance with seasonal cycles and geographic proximity, may lead to an ecological paradox, undermining the very environmental goals that the MD seeks to promote [124].

A second area of concern pertains to the semantic and commercial appropriation of the “Mediterranean diet” label by the food industry. In many cases, highly processed foods with poor nutritional quality are marketed as “Mediterranean,” capitalizing on the model’s positive reputation. This process of commodification and dilution not only creates confusion among consumers but also weakens the effectiveness of public health campaigns aiming to promote the authentic Mediterranean pattern [125].

From a socioeconomic standpoint, MD can present barriers to accessibility, both financial and logistical. The perceived high cost of fresh, local, and organic products, core elements of the diet, constitutes a significant obstacle for low-income populations. Additionally, the lack of time, culinary skills, and supportive infrastructures (e.g., local markets, adequately equipped home kitchens) may hinder consistent adherence to MD, particularly in urban contexts and among younger generations [126].

Finally, it must be emphasized that nominal adherence to Mediterranean dietary guidelines does not automatically translate into environmental sustainability. Simply following nutritional recommendations, without careful consideration of product origin, production methods, and distribution channels, may negate a substantial part of the expected benefits. For instance, consuming foods sourced from global supply chains or produced using high-impact agricultural practices can result in a marked disconnect between the theoretical model and its practical implementation [127].

In summary, a mature and scientifically grounded approach to promoting the Mediterranean diet must involve a critical, contextualized, and systemic evaluation, capable of acknowledging its limitations while integrating environmental, social, and economic criteria into its definition and application. Only through such a perspective can the integrity and effectiveness of MD be preserved as a practical tool for transitioning toward truly sustainable food systems.

## 6. Decline of Adherence to the Mediterranean Diet: Causes, Implications, and Counterstrategies

In recent years, a worrying decline in adherence to the MD has been seen, even in the countries of the Mediterranean basin, where this dietary model has deep origins and cultural roots. MD has been celebrated for decades for its beneficial effects on human health and for its environmental sustainability. However, although the evidence linking MD to improved health outcomes is extensive, it is important to critically acknowledge certain limitations of the existing studies. Many investigations rely on self-reported dietary assessments, which may introduce recall or social desirability biases, and the definition of “Mediterranean diet” often varies across studies and populations, affecting comparability [128]. Furthermore, effect sizes, while generally favorable, are heterogeneous: large meta-analyses indicate approximately 20–30% relative risk reduction for cardiovascular diseases and all-cause mortality, but smaller or non-significant effects for certain metabolic and cancer outcomes [129,130]. These discrepancies highlight the importance of contextual and behavioral factors, as well as the need for long-term, population-specific research to fully quantify the causal impact of MD on health.

Historically, MD developed as a largely plant-based, seasonal, and locally sourced dietary pattern, deeply rooted in the cultural, economic, and environmental context of Mediterranean populations. Its principles reflected not only nutritional wisdom but also the realities of subsistence agriculture, seasonal food availability, and traditional culinary practices transmitted across generations. Contemporary socio-economic and cultural transformations, such as urbanization, globalization, widespread availability of ultra-processed foods, time constraints, and changing family structures, have significantly altered this historical context. As a result, adherence to the traditional MD is challenged today, highlighting the need to adapt its principles to modern lifestyles while preserving its health and sustainability benefits.

In Italy, a study conducted by CREA (Research Center for Food and Nutrition) on a sample of about 3000 people found that only 5% of Italian adults fully adhere to MD, while the majority show a moderate or low level of adherence. Particularly alarming is the situation among younger generations: a significant reduction is observed in the consumption of key foods of the Mediterranean model, such as fruits, vegetables, legumes, and whole grains, accompanied by an increase in the consumption of meat and ultra-processed foods. For example, 9% of children and adolescents declare that they never consume vegetables, and 7% do not eat fruit, while 47% consume more than three portions of meat per week, a frequency well beyond Mediterranean nutritional recommendations. Even among university students, although the average adherence is around 72%, a decreasing trend is recorded, with a low presence of fundamental plant-based foods in the daily diet [131].

Recent comparative studies show that this trend is not unique to Italy. Similar declines in adherence to the Mediterranean diet have been reported in Spain, Greece, and Cyprus, particularly among younger generations, reflecting a broader Mediterranean pattern of “nutritional Westernization” [122]. As summarized in Figure 2, the main drivers of this decline include urbanization, high perceived costs, the influence of social media and fast-food culture, and the erosion of traditional culinary knowledge and motivation to cook fresh foods. Rather than isolated behaviors, these factors represent systemic changes in food environments and cultural values, making younger generations increasingly distant from the dietary patterns traditionally rooted in the Mediterranean lifestyle [132,133]. Addressing these barriers requires integrated educational and policy actions that reconnect MD with modern social realities while preserving its cultural and nutritional identity.

The causes of this decline are complex and multifactorial. One of the main factors is represented by the increasing urbanization and lifestyle changes. Modernization has led to an increasingly hectic daily life, which reduces the time available for meal preparation and encourages the consumption of ready-made, prepackaged foods with high energy content, often poor in essential nutrients. This “nutritional transition” is accompanied by the global spread of “Westernized” dietary models, dominated by industrial products rich in sugars, saturated fats, and salt. A significant role in this process is played by economic factors, with the economic crisis and the reduction in disposable income accelerating the abandonment of MD, particularly among the elderly and people with fewer economic resources. Young people in particular perceive MD as “too expensive” (50% of youths) and “too time-consuming” (38%), while in other age groups these percentages fall, respectively, to 42% and 27% [125].

The food industry significantly contributes to this phenomenon through misleading marketing strategies, with numerous ultra-processed products advertised as “Mediterranean” despite having little to do with the real MD [134]. A study found that between 2011 and 2020, as many as 1219 food advertisements used the semantic field “Mediterranean” as part of their discursive strategy, often to promote products with poor or null nutritional value [125].

Added hindering factors include cognitive and motivational aspects, such as poor knowledge of the health benefits of Mediterranean foods, the perceived complexity of dietary plans, and the lack of nutritional education. Food neophobia, that is, reluctance to try new or unfamiliar foods, has proven to be a significant negative predictor of adherence to MD, especially among young people. Sensory and hedonic factors play an important role, with many individuals reporting not liking the taste of key foods such as legumes, having difficulty giving up preferred foods, or feeling little sensory appeal towards MD components [135]. The loss of culinary traditions is a critical element, with the interruption of intergenerational transmission of food knowledge that traditionally took place through women, custodians of culinary techniques and seasonal rhythms. Socio-cultural changes have deeply altered food consumption patterns, with negative influence from family members, work pressures, and irregular schedules hindering the adoption of Mediterranean eating habits [136]. Food globalization, intensified by tourism and the spread of fast-food chains, has led to a standardization of tastes towards so-called “junk food,” particularly clear in coastal areas.

The advent of the digital age has introduced new challenges, with social media, despite its educational potential, often promoting contradictory nutritional information and not having a proven significant impact in determining positive changes in daily food choices. Food delivery services and digital platforms, while offering some Mediterranean options, tend to favor ultra-processed foods and quick preparations that contrast with the principles of MD [137].

Climate change appears as a growing threat to the sustainability of MD, with rising temperatures and water scarcity compromising the production of key foods such as olives, grapes, and various vegetables. Work-related factors contribute to the decline, with stressful work environments, long hours, and the lack of Mediterranean food options in corporate canteens pushing towards suboptimal food choices. The lack of culinary skills and appropriate equipment for preparing fresh meals represents a significant obstacle, particularly among younger generations who have not developed the necessary skills to cook according to Mediterranean principles [83,138].

From a geographic point of view, adherence to MD varies significantly within the same Italian territory. Regions such as Emilia-Romagna, Lazio, Sicily, and Sardinia show relatively higher levels of adherence, while areas such as the Northeast and Campania show lower values, often correlated with lower levels of nutritional literacy [139].

Beyond the negative effects on public health, the decline in adherence to MD has relevant environmental implications. The progressive abandonment of a dietary regime traditionally based on local and seasonal plant-based foods leads to an increase in ecological impact, mainly linked to higher consumption of meat and ultra-processed foods, notoriously more demanding in terms of natural resources and greenhouse gas emissions.

Economic and political factors also play a central role in shaping adherence to MD, particularly through their impact on education and knowledge transmission. Public policies, funding for school-based nutritional programs, and community initiatives determine the reach and quality of food education, influencing the ability of individuals to acquire culinary skills and nutritional literacy [140]. Socioeconomic disparities limit access to educational resources, cooking classes, and local food markets, disproportionately affecting populations with low income or in rural areas [141]. Political support for campaigns promoting traditional dietary knowledge, culinary heritage, and sustainable food practices can strengthen adherence, while a lack of institutional investment contributes to the erosion of intergenerational knowledge transfer. In this context, low adherence to MD is not only a matter of personal choice but also reflects broader systemic inequalities in education, public health policies, and economic access to Mediterranean foods.

To counteract this phenomenon and promote a sustainable food transition, it is necessary to intervene on multiple levels. Scientific and health institutions, such as the Italian Society of Human Nutrition (SINU), have developed new versions of the Mediterranean food pyramid, adapted to the contemporary context, aiming to offer practical guidance for a balanced, healthy, and environmentally respectful diet. These initiatives place particular emphasis on food education, especially in schools, and on the importance of public policies that encourage conscious and accessible food choices [142].

At the same time, strategies to promote MD must recognize the central role of consumers as agents of change. An informed and aware population, capable of making food choices based on health and sustainability criteria, can positively influence the entire agri-food system. In this sense, multisectoral commitment, involving institutions, educators, media, producers, and distributors, is fundamental to creating food environments favorable to a return to traditional and sustainable models.

**Figure 3:**
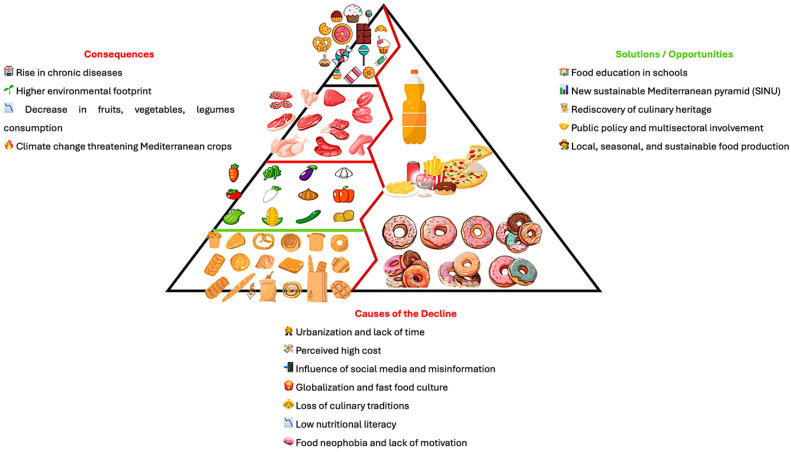
The main causes of the decline in adherence to the Mediterranean Diet. Principal consequences (**Left**) and major solutions/opportunities (**Right**).

In conclusion, the decline in adherence to MD is the result of a series of socio-economic, cultural, and behavioral transformations deeply changing the eating habits of Mediterranean populations. This phenomenon is a challenge for public health and environmental sustainability but at the same time offers an opportunity: that of rethinking food systems, promoting the rediscovery and adaptation of the Mediterranean food heritage to the needs of the modern world, without betraying its fundamental principles (Figure 3).

## 7. Conclusions

MD today is much more than a mere dietary regimen: it is a systemic model that integrates health, culture, environmental sustainability, and social equity. Its nutritional structure, centered on fresh, seasonal plant-based foods, extra virgin olive oil, and moderate consumption of animal products, has proven highly effective in preventing chronic degenerative diseases, promoting public health, and reducing overall mortality [123]. However, the true value of MD lies in its holistic approach that links human well-being with ecological balance and social cohesion.

Scientific evidence reviewed in this article confirms the MD's crucial role in the transition towards sustainable food systems. Compared to Western dietary models, MD is associated with significantly lower greenhouse gas emissions, a smaller water footprint, reduced energy consumption, more efficient land use, and positive impacts on biodiversity conservation and soil regeneration. Furthermore, traditional agricultural practices linked to the MD, such as polyculture, crop rotation, and the use of perennial crops, promote carbon sequestration and agroecological resilience [81].

From a socio-cultural perspective, MD is a rich intangible heritage of knowledge, practices, and values that foster conviviality, intergenerational transmission, and local identity. The act of sharing meals, the central role of women in culinary traditions, and the preservation of seasonal rhythms all contribute to a food culture that nurtures not only physical but also psychological and emotional well-being [50].

Despite these well-documented benefits, a troubling decline in adherence to the MD is being observed, even in Mediterranean countries. Younger generations are increasingly drawn to globalized food models dominated by ultra-processed, calorie-dense, and nutrient-poor products. This trend is driven by complex factors, including time constraints, the rise in food delivery services, the erosion of culinary skills, the perceived high costs of traditional diets, and misleading marketing practices that dilute the true essence of MD [131].

To reverse this trend, a systemic, multisectoral, and multilevel response is needed. Public institutions, academia, schools, media, and the food industry must collaborate to promote supportive food environments, sustainable farming practices, nutrition education, and economic accessibility to healthy, local food. Reconnecting food with place and culture is essential to reaffirm MD as a model of ecological and civic responsibility.

In addition to evidence-based findings, we also offer reflective considerations on practical strategies to improve accessibility and implementation of the MD, particularly in urban or economically disadvantaged communities. These reflections, while informed by the literature, are not themselves direct outcomes of empirical studies and are provided to contextualize how MD principles can be translated into policy, educational, and community interventions.

To improve accessibility of MD, particularly in urban or economically disadvantaged communities, a combination of policy, educational, and structural interventions is essential. Public policies could include subsidies for local and seasonal produce, incentives for urban agriculture and farmers’ markets, and support for community gardens that provide affordable fresh fruits and vegetables. Nutrition education programs, integrated into schools, workplaces, and community centers, can raise awareness of the health, environmental, and cultural benefits of MD while teaching practical cooking skills. Partnerships with local retailers and food delivery services could prioritize affordable Mediterranean food options and promote culturally tailored meal kits or vouchers. Additionally, fostering social initiatives, such as community kitchens and cooperative food networks, can enhance access, reduce costs, and strengthen communal engagement around healthy, sustainable eating. These measures collectively ensure that the benefits of MD are not limited to affluent or rural populations but are equitably accessible to all segments of society.

Looking ahead, MD should be viewed not only as a legacy to preserve but also as a flexible and scalable platform adaptable to diverse geographic and cultural contexts. Its scientific foundation and adaptability make it an ideal candidate to inform global sustainable food policies. Integrating the Mediterranean model into public health initiatives, climate change strategies, and sustainable development agendas, such as the UN’s 2030 Agenda, could yield substantial long-term benefits.

In conclusion, reviving the Mediterranean diet means embracing a deep human, ecological, and cultural vision of food. It means reclaiming a balanced relationship with nourishment, time, others, and the environment. It means building a food future where health, social justice, and environmental respect are not separate goals but interconnected pillars of an integrated vision. With its rich cultural heritage and scientific robustness, the MD is still one of the most effective tools we have for guiding this transformation.

## Figures and Tables

**Table 1:** Comparative summary of the main differences between the MD and the Western diet. MJ/year = megajoules per year; ha/year = hectares per year.

Indicator	Mediterranean Diet	Western Diet	Mediterranean Advantage
CO_2_ emissions (kg/day)	2.3–5.08	7.4–8.5	−30%/−50%
Water consumption (L/day)	3243–4000	4347–5000+	−25%
Food waste	Low	High	−50%
Energy use (MJ/year)	5250	7300	−28%
Land use (ha/year)	2000	3300	−40%
Biodiversity	High	Low	+Variety, +Resilience

**Table 2:** List of articles included in this review.

Author(s) and Year	Title/Focus	Main Conclusions
Han et al., 2022 [80]	Carbon Footprint Research Based on Input-Output Model	Growing research on carbon footprint measurement; global scientometric trends.
Sáez-Almendros et al., 2013 [81]	Environmental Footprints of Mediterranean vs. Western dietary Patterns	MD has a lower carbon and ecological footprint than the Western diet.
Vidal et al., 2015 [82]	Carbon Footprint of Patient Diets in Spanish Hospital	More plant-based diets reduce carbon footprint; MD is applicable in hospital settings.
Castaldi et al., 2022 [83]	Positive Climate Impact of Mediterranean Diet	MD is climate-friendly; divergence in some Mediterranean countries reduces benefits.
Barthelmie, 2022 [84]	Dietary Meat and Animal Products on GHG Footprints	Diets rich in meat generate up to 5x more emissions; reducing meat improves sustainability.
Munoz et al., 2015 [85]	Greenhouse vs. Open-field Tomato Production	Greenhouse cultivation increases energy use; local open-field production is preferred.
Lorca-Camara et al., 2024 [86]	Environmental and Health Sustainability of MD	MD is both healthy and sustainable; integration of health and environmental outcomes.
Conrad et al., 2025 [87]	Are Healthier Diets More Sustainable?	Higher quality diets tend to be more sustainable; variation among indexes.
Micheloni et al., 2025 [88]	Sustainability of the Mediterranean Diet	MD reduces GHG and footprint; provides nutritional benefits.
Niamir-Fuller, 2016 [89]	Sustainability in Livestock	Sustainable livestock management reduces environmental impacts, relevant for protein choices in MD.
Lovarelli et al., 2016 [90]	Water Footprint of Crop Productions	Crop water use analysis; supports sustainable MD planning.
Vanham et al., 2021 [91]	Which Diet Has the Lower Water Footprint in Mediterranean Countries?	MD with more plant-based foods has a lower water footprint.
Bordoni, 2023 [92]	Water Footprint of Italian MD	MD shows lower water consumption compared to the Western diet.
Mekonnen & Hoekstra, 2012 [93]	Global Water Footprint of Farm Animal Products	Animal products require much more water; plant-based MD is advantageous.
Vanham et al., 2020 [95]	Treenuts and Groundnuts in EAT-Lancet	Water use concerns; highlights the importance of sustainable nut consumption in MD.
Aboussaleh et al., 2017 [96]	Mediterranean Food Consumption Patterns	MD has a low environmental impact and health benefits.
Yang et al., 2024 [97]	Diversifying Crop Rotation	Crop rotation increases food production, reduces GHG, and improves soil health.
Santonocito et al., 2024 [98]	Mediterranean Food By-Products	Valorization of by-products enhances sustainability and nutritional value.
Nucci et al., 2025 [99]	Mediterranean Diet and Household Food Waste	MD reduces household food waste.
Annunziata et al., 2019 [100]	Sustainability of Italian Families’ Food Practices	MD with local/organic foods increases sustainability.
Abderrrezag et al., 2025 [101]	Nutraceutical Potential of MD Agri-Food Waste	By-product valorization reduces waste and enhances health benefits.
Balan et al., 2022 [102]	Metabolic Food Waste	MD can reduce food waste and related environmental impacts.
Pairotti et al., 2015 [103]	Energy Consumption and GHG of MD	MD consumes less energy and emits less GHG compared to Western diet.
Nijdam et al., 2012 [104]	Land Use and Carbon Footprints of Animal Products	Meat has a higher footprint; plant-based substitutions reduce impact.
Álvarez-Álvarez et al., 2024 [109]	Impact of MD Promotion on Environmental Sustainability	Promoting MD reduces environmental impacts; effective policy tool.
Willett et al., 2019 [110]	EAT-Lancet Commission	Healthy and sustainable diets, MD as a global model for sustainable food systems.
Blas et al., 2019 [111]	MD vs. Current Food Patterns in Spain	MD has a lower water and nutritional footprint.
Carranca et al., 2022 [112]	Enhancing Carbon Sequestration in Mediterranean Agroforestry	Agroforestry increases carbon sequestration; MD + sustainable practices are beneficial.
Mattas et al., 2023 [113]	Biodiversity and Diet through MD	MD supports agricultural biodiversity.
Korpelainen, 2023 [115]	Home Gardens in Promoting Biodiversity and Food Security	Home gardens enhance biodiversity and food security; complement MD.
Álvaro-Fuentes & Paustian, 2011 [119]	Soil Carbon Sequestration in Semiarid Mediterranean	Soil management increases carbon sequestration; MD integration is beneficial.
Kan et al., 2022 [120]	Soil Organic Carbon Stability and No-till	No-till farming stabilizes soil carbon; supports sustainable MD practices.
Altieri et al., 2015 [121]	Agroecology and Climate-Resilient Farming	Agroecology improves resilience and carbon sequestration, compatible with MD.

**Table 3:** Comparative summary of structural limitations of MD.

Aspect	Description	Implications
Water Footprint	Some typical foods require high water consumption, especially in contexts with water scarcity and intensive agriculture.	It can create an ecological paradox, undermining the environmental goals of the MD if production is not managed sustainably.
Commercialization	The term “Mediterranean Diet” is often used to market highly processed foods with poor nutritional quality.	Consumer confusion and weakening of public health campaigns promoting the authentic model.
Socioeconomic Barriers	Perceived high cost of fresh, local, and organic products; lack of time, culinary skills, and adequate infrastructure.	Limited access for low-income groups and difficulties in adherence, especially in urban contexts and among younger generations.
Nominal Adherence	Following nutritional guidelines without considering product origin, production methods, and supply chains can negate expected environmental benefits.	Risk of disconnect between theoretical model and real practice, compromising the diet’s actual sustainability.

## Data Availability

No new data were created or analyzed in this study. Data sharing is not applicable to this article.

## Abbreviations

CO_2_eq	CO_2_ equivalent
EVOO	Extra virgin olive oil
MD	Mediterranean diet
SCFAs	Short-chain fatty acids
SOC	Soil organic carbon
WF	Water footprint
